# Gene expression profiles in *Rana pirica* tadpoles following exposure to a predation threat

**DOI:** 10.1186/s12864-015-1389-4

**Published:** 2015-04-02

**Authors:** Tsukasa Mori, Yukio Yanagisawa, Yoichiro Kitani, Manabu Sugiyama, Osamu Kishida, Kinya Nishimura

**Affiliations:** Department of Marine Science and Resources, Nihon University College of Bioresource Sciences, Kameino 1866, Fujisawa, 252-0880 Japan; Department of Liberal Art, Nihon University College of Bioresource Sciences, Kameino 1866 Fujisawa, 252-0880 Japan; Teshio Experimental Forest, Field Science Center for Northern Biosphere, Hokkaido University, Horonobe, Hokkaido 098-2943 Japan; Graduate School of Fisheries Sciences, Hokkaido University, Hakodate, 041-8611 Japan

**Keywords:** Cross-species microarray, Tadpoles, Discriminant analysis, Predator-induced

## Abstract

**Background:**

*Rana pirica* tadpoles show morphological changes in response to a predation threat: larvae of the dragonfly *Aeshna nigroflava* induce heightened tail depth, whereas larval salamander *Hynobius retardatus* induce a bulgy morphology with heightened tail depth. Although both predators induce similar tail morphologies, it is possible that there are functional differences between these tail morphs.

**Results:**

Here, we performed a discriminant microarray analysis using *Xenopus laevis* genome arrays to compare tail tissues of control and predator-exposed tadpoles. We identified 9 genes showing large-scale changes in their expression profile: *ELAV*-*like1*, *methyltransferase like 7A*, *dolichyl-phosphate mannosyltransferase*, *laminin subunit beta-1*, *gremlin 1*, *BCL6 corepressor-like 1*, and three genes of unknown identity. A further 80 genes showed greater than 5 fold differences in expression after exposure to dragonfly larvae and 81 genes showed altered expression after exposure to larval salamanders. Predation-threat responsive genes were identified by selecting genes that reverted to control levels of expression following removal of the predator. Thirteen genes were induced specifically by dragonfly larvae, nine others were salamander-specific, and sixteen were induced by both. Functional analyses indicated that some of the genes induced by dragonfly larvae caused an increase in laminins necessary for cell adhesion in the extracellular matrix. The higher expression of *gremlin 1* and *HIF1a* genes after exposure to dragonfly larvae indicated an in vivo hypoxic reaction, while down-regulation of *syndecan-2* may indicate impairment of angiogenesis. Exposure to larval salamanders caused down-regulation of *XCIRP-1*, which is known to inhibit expression of adhesion molecules; the tadpoles showed reduced expression of *cα(E)-catenin*, small muscle protein, *dystrophin*, and *myosin light chain* genes.

**Conclusion:**

The connective tissue of tadpoles exposed to larval salamanders may be looser. The differences in gene expression profiles induced by the two predators suggest that there are functional differences between the altered tail tissues of the two groups of tadpoles.

**Electronic supplementary material:**

The online version of this article (doi:10.1186/s12864-015-1389-4) contains supplementary material, which is available to authorized users.

## Background

Phenotypic plasticity is the ability to produce different phenotypes under different environmental conditions and to respond to changes in environmental conditions [1-3]. This phenomenon has long been studied by evolutionary biologists interested in its adaptive significance [4,5]. One particular type of phenotypic plasticity is that displayed as an inducible defense, which is stimulated directly by cues associated with a predation threat [6-12]. Predator-induced phenotypic plasticity in anuran tadpoles has been extensively studied. Anuran tadpoles exhibit a range of inducible morphological changes, such as heightened tail depth, in the presence of a threat by various types of pond dwelling predator such as dragonfly larvae [13-15]. Tadpoles with a heightened tail depth phenotype show higher survival rates when dragonfly larvae are present [16,17]. *Rana pirica* tadpoles display a unique bulgy morph when exposed to their main predator, larval salamander *Hynobius retardatus* [10]. The inducible bulgy morphology is believed to be an evolutionary defense against the gape-limited *H. retardatus* larvae under an intimate predator–prey relationship [18-21]; the bulgy morph is only induced by a predation threat from larval salamanders and functions to prevent the tadpoles from being swallowed [10,17].

*R. pirica* presumably evolved phenotypic plasticity in tadpoles as a defense against specific predators: the inducible bulgy body with heightened tail against larval salamanders and the heightened tail morph against the larvae of the dragonfly *Aeshna nigroflava*. However, dragonfly larvae are top predators in natural ponds [22], and the survival rate of *Rana pirica* tadpoles with the salamander-induced bulgy morph is lower than that of tadpoles with the dragonfly-induced heightened tail when exposed to predation by dragonfly larvae. Furthermore, bulgy morph tadpoles have the same survival rate as non-induced tadpoles when placed with dragonfly larvae. Tadpoles with the dragonfly-induced higher tail morphology are less vulnerable to predation by larval salamanders than non-induced tadpoles, indicating that the higher tail phenotype has adaptive advantages compared to other phenotypes under conditions of salamander and dragonfly predation [17]. Moreover, in the presence of dragonfly risk cues, tadpoles with a bulgy morph and heightened tail induced by an earlier exposure to a salamander reduce only the bulgy body but retain the heightened tail [23].

Thus, the tadpoles can regulate their morph according to differences in predator cues; this behavior offers a valuable system for investigating switching mechanisms in adaptive phenotypic plasticity against predators. The evolutionary aspects of these complex phenotypic changes have been addressed in a number of studies over the last decade [20,24-27].

Although some studies have identified genetic variation and geographic differentiation in anuran tadpoles with respect to inducible anti-predation defenses, and have shown that these traits are heritable [20,27], we have very little understanding of the genetic mechanisms involved in the morphogenetic alterations associated with these defense traits.

We previously conducted cDNA subtraction and species-specific microarray analyses of epithelial tissues from *R. pirica* tadpoles, both bulgy morph and non-bulgy morph [28], and identified key genes relating to morphogenetic changes [29]. A larger functional species-specific microarray (3 k array) was prepared in the previous study [29] and used in induction-reversion experiments to analyze mRNAs extracted from the facial tissues of tadpoles. These analyses identified a novel uromodulin-like gene, *pirica*, that was increasingly up-regulated as the period of exposure to larval salamanders lengthened. The pirica protein was found to contain a zona pellucida domain similar to proteins that function to control water permeability; the *pirica* gene was shown to be expressed in the superficial epidermis of the tadpole skin [29]. We also demonstrated that water retention in the connective tissue and maintenance of a constant osmotic pressure were important factors for bulgy morph formation, supporting the interpretation that predator-induced expression of *pirica* in the skin causes retention of absorbed water [30]. The immuno-related proteins hyaluronic acid, histone H3 and 14-3-3 zeta were the most abundant constituents of the liquid aspirated from the connective tissue. These findings suggested that formation of the bulgy morph might also require activation of the innate immune system [30].

We concluded from our previous studies that evolution of the inducible bulgy morphology against the gape-limited *H. retardatus* larvae involved changes to the control of body water dynamics and that some key genes were involved in production of the bulgy body. As mentioned above, the heightened tail morph induced by dragonfly larvae appears to provide greater protection against predators, although *Rana pirica* tadpoles are able to adopt another morphology in response to an intermediate predator, i.e., larval salamanders. This ability to switch response suggests expression of both a common set of genes against predators and also a predator-specific set of genes. In the present study, we sought to answer two particular questions: first, do the bulgy morph and the heightened tail morph induced by salamander and dragonfly larvae, respectively, differ with respect to gene expression patterns; second, which genes are induced by all predators and which are predator-specific?

## Methods

### Ethics statement

All animal experiments were conducted by trained personnel in accordance with the guidelines of the Animal Care Committee, Nihon University.

### Experimental animals and design of the microarray

Eggs of *R. pirica* and larvae of the dragonfly *Aeshna nigroflava* and the salamander *H. retardatus* were collected from a pond in Hokkaido, Japan, and placed in separate 12 liter aquaria. After hatching, *R. pirica* tadpoles were fed rabbit chow *ad libitum*. Larval *H. retardatus* and *A. nigroflava* were fed small *R. pirica* tadpoles *ad libitum*. Water in all aquaria was changed every second day. The experiment was conducted in a laboratory at 18°C, using a natural day/night (about 14/10 hours) regime. Experiments were performed using 4 liter aquaria (29 × 16.5 cm surface area, height 9 cm) each containing 2 liters of aged tap water. Fifty 10-day-old tadpoles of similar sizes (mean body length = 7.90 ± 0.38 mm (standard deviation), n = 48) were randomly chosen from the holding tank and placed in each aquarium. The water in all aquaria was changed every second day throughout the experimental period.

**Figure 1:**
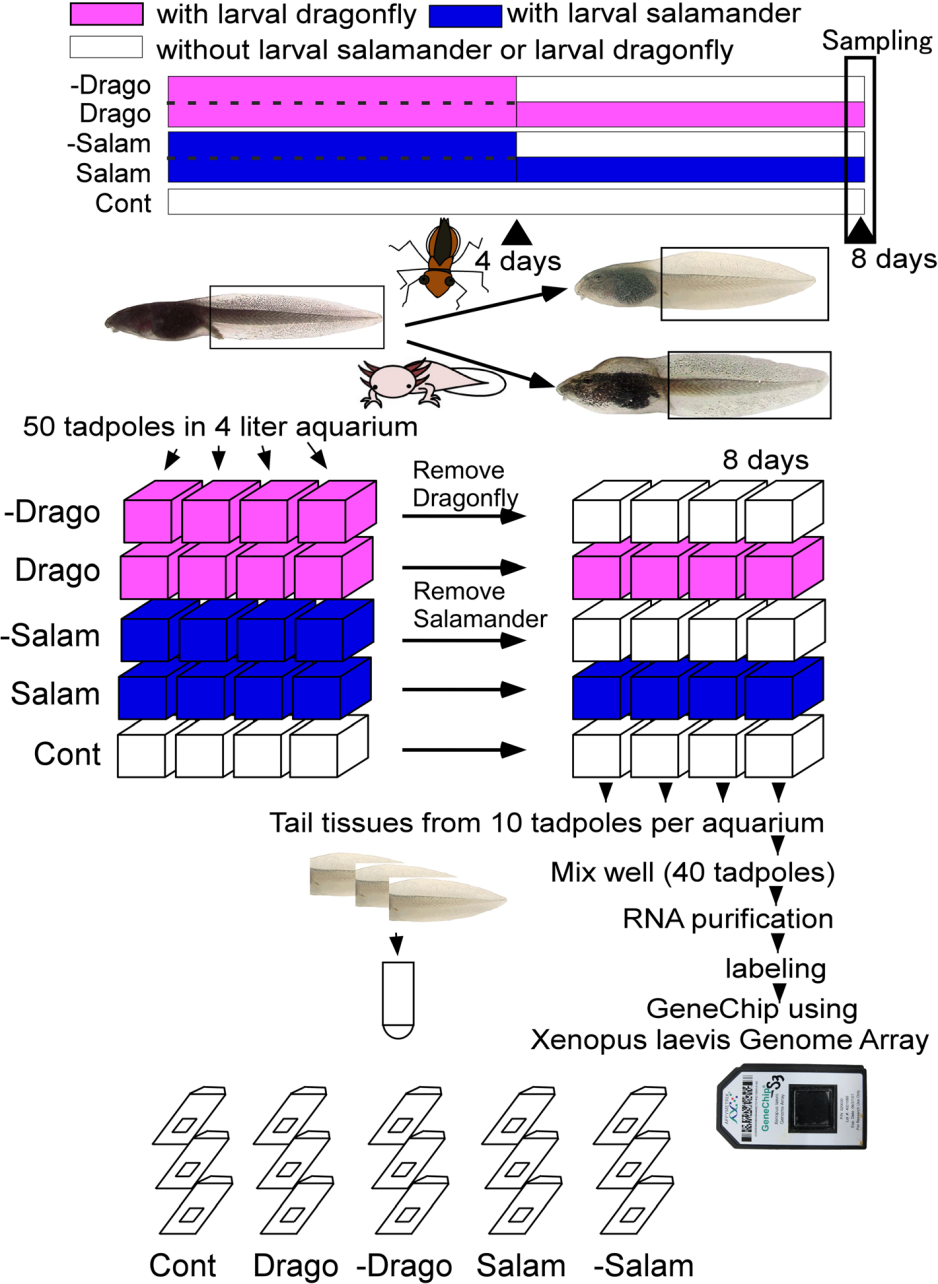
**Experimental design showing control, continuous exposure treatment to dragonfly larvae or larval salamanders, and removal of the predation threat to allow the tadpoles to recover.** These five treatment groups were used to produce the RNAs used in the microarray analysis with the Xenopus genome array. In the group with continuous exposure to predators (Drago or Salam), the tadpoles were under predation threat for the full 8 days. In the groups exposed for a limited period and then allowed to recover (−Drago or −Salam), tadpoles were initially kept with dragonfly larvae or larval salamanders for 4 days to produce the predator-induced phenotype; after 4 days, the predation threat was removed and the tadpoles were allowed to revert to their normal phenotype for 4 days. The control group of tadpoles was not exposed to a predation threat. Four replicate groups were used for each treatment; tadpoles were sampled on day 8. Tail tissues from the tadpoles were used for RNA extraction for the microarray analysis. The microarray analysis was performed in triplicate. Abbreviations: Cont, control; Drago, 8 day dragonfly exposure; Salam, 8 day salamander exposure; −Drago, 4 day exposure to dragonfly threat and 4 day recovery; −Salam, 4 day exposure to salamander threat and 4 day recovery.

The protocol for experiments involving continuous or short-term exposure of tadpoles to a predator is summarized in Figure 1. Four predator exposure conditions were used, along with a non-exposed control: 20 aquaria were set up for predation-exposed and control tadpoles (we also set up a further 4 aquaria as a backup only for the salamander treatments). Thus, each treatment group had 4 replicate aquaria. The dragonfly and salamander predation experiments were initiated by the introduction of either a large dragonfly larva in a cage or three larval salamanders (about 18 mm length). In two groups, the predators were removed after 4 days and the tadpoles were allowed to revert to a normal phenotype (−Drago and −Salam). At 8 days, we collected tail tissue samples from tadpoles in the aquaria of each experimental group, and performed the microarray analysis using these triplicate samples.

To minimize loss of tadpoles in the salamander treatment groups (Salam), the larval salamanders were replaced daily by others that had been kept in a holding tank with sufficient *R. pirica* tadpoles to allow easy feeding. The replacement predators were chosen randomly from each holding tank. Every second day, we counted the number of tadpoles in each aquarium.

For the microarray, RNAs were extracted and prepared from 10 tadpoles from each 4 liter aquaria. Therefore, a total of 40 tadpoles were used for each treatment group. Tail tissue was dissected from each tadpole and cut into small pieces; the tissues from all 40 tadpoles of each treatment group were combined and used for RNA extraction. The extracted RNA from each group of 40 tadpoles served as replications for each comparative experiment.

### RNA extraction

The tail tissue samples from tadpoles in each treatment group were placed in a single tube containing RNAlater™ (QIAGEN RNA stabilization reagent). The samples were stored at −80°C until RNA extraction. Total RNA was concentrated using an RNeasy Midi Kit® according to the manufacturer’s protocol. Total RNA purity was determined by measuring the *A*_260_/*A*_280_ absorbance ratio and by visualizing the 18S and 28S ribosomal RNA using gel electrophoresis. RNA samples that had an *A*_260_/*A*_280_ ratio between 1.7 and 2.1 and passed a visual examination of 18S and 26S ribosomal RNA were further processed for the microarray analysis.

### Cross-species microarray

Due to the lack of a large-scale microarray from *R. pirica,* we chose to make use of a cross-species protocol. Microarrays from *Xenopus laevis* and *X. tropicalis* are commercially available (Affymetrix). A comparison of e-values from microarrays of the two species after probing with *R. pirica* mRNAs indicated there were no statistically significant differences (see Additional file 1 and Additional file 2: Figure S1). We made use of the *X. laevis* microarray for the analyses of predator-induced gene expression in *R. pirica* reported here.

Samples were prepared for microarray analysis according to Affymetrix GeneChip® protocols. An aliquot containing 5 μg total RNA was converted to double-stranded cDNA with an Affymetrix One-Cycle cDNA Synthesis kit (Affymetrix). Biotin-labeled cRNA was generated from the cDNA samples by *in vitro* transcription with T7 RNA polymerase using an Affymetrix IVT Labeling kit (Affymetrix). The biotin-labeled cRNAs were fragmented to an average size of 35–200 bases by incubation at 94°C for 35 min in the fragmentation buffer provided in the Affymetrix sample cleanup kit. Each fragmented sample (15 μg) was hybridized for 16 h at 45°C onto individual *Xenopus laevis* genome arrays, which enable analysis of 15611 transcripts. The gene chips were washed and stained in an Affymetrix Fluidics Station 450 according to the GeneChip®Expression Analysis Technical Manual. The chips were scanned with the GeneChip®Scanner 3000 (Affymetrix), and the data was imported into the GeneChip Operating software (GCOS v1.2). The microarray data analysis was carried out using Agilent GeneSpring v11.5, and the data was normalized using the MAS5 summarization algorithm and baseline to median of all samples by baseline option. The data discussed in this publication have been deposited in the NCBI Gene Expression Omnibus (GEO) and are accessible through GEO Series accession number GSE33250.

### Analysis of the microarray data

In this study, it was not appropriate to use normal data processing procedures, such as false discovery rate and other strict statistical methods for screening, due to the low signal intensities obtained in the cross-species hybridization [31]. Thus, to identify genes that were influenced by the predation treatments among the 15611 genes on the microarray, we assumed that the response of each gene was independent of that of other genes. It was necessary to repeat the 15611 independent statistical tests, e.g., analysis of variance, to identify sets of genes that gave different population mean responses depending upon the level of the factor. However, if more than 10000 independent statistical tests with a 5% significance level are performed, then the probability that at least one of the tests will be significant is almost 1. Therefore, we performed multiple comparison tests. As 15611 independent statistical tests, with a family-wise error rate of 5% (i.e., the probability that at least one of the statistical tests will be significant is 5% when all the population means are the same), were performed, then the significance level of each test is 1–(1–0.05)^1/15611^ = 0.00032857%, i.e. 0.00032857%. The family-wise error rate has also been termed the experiment-wise error rate (see Hsu, 1996 [32]). Usually, the number of tests in a multiple comparison test is up to about 20, or possibly 100 in an extreme case. The number of statistical tests corresponding to the number of genes is over 10000, which is an extremely large number. If the family-wise error rate is 5%, then the significance level for each test of 0.00032857% is too low to permit detection of differences among population means. However, if we use a family-wise error rate of 90% for each of the 15611 tests, then a significance level of 1–(1–0.90)^1/15611^ = 0.0001474867 (or 0.01474867%) is obtained. As a compromise, we performed ANOVA with a significance level of 0.01474867% to identify a set of genes from the total of 15611 on the microarray that might be influenced by the predation treatments.

The second step in the data analysis was validation of the selection of the gene set in the first stage of analysis. To achieve this validation, we applied a discriminant analysis to obtain a function or functions that differed among treatment groups. The canonical discriminant functions are calculated by maximizing a fraction, the projection of a linear function of the variance covariance matrix of between groups to that of the variance covariance matrix within the groups, i.e. maximizing the separation of the groups. As another validation step, we applied a hierarchical cluster analysis; we chose single linkage with Euclidean distance, group average with Euclidean distance and Ward’s method with squared Euclidean distance. Single linkage is a suitable method to identify outliers and the other methods are suitable to identify like clods. The question of which method should be used depends on the definition of preferred clusters (see [33]). However, if clusters are clearly separated, i.e. within clusters are concentrated on their center, and between clusters are clearly distinct, then whichever method of cluster analysis is used, we obtain the same conclusion; this shows the robustness of the selection of the gene set at the first analytical step.

We compared predator-specific and non-specific gene expression patterns in tadpoles allowed to recover from exposure dragonfly or larval salamanders. In this analysis, we performed data mining to identify changes in expression greater than five-fold compared to the controls. The fold change calculation was performed for each probe set by comparing the mean value for the predator treatments samples to the mean value for the control samples.

### Pathway analysis of genes selected by discriminant analysis

Functional information on the nine genes selected by the discriminant analysis (Table 1) was used in an Ingenuity Pathway Analysis (IPA, Qiagen).

**Table 1:** **Summary of the nine genes selected by discriminant analysis**

**Gene no**	**Probe set ID**	**[−Salam]/[cont]**	**[−Drago]/[cont]**	**[Salam]/[cont]**	**[Drago]/[cont]**	**Gene symbol**	**Gene title**	**Accession no.**	**Gene ontology (GO ID : GO Term)**
		**Fold change**	**Fold change**	**Fold change**	**Fold change**				
		**Regulation**	**Regulation**	**Regulation**	**Regulation**				
①	Xl.5177.1.S1_at	−3.3129547	1.3858212	1.3897077	1.2546494	elavl1-a	ELAV-like 1	NM_001090609	0000166: nucleotide binding
		down	up	up	up				0003676: nucleic acid binding
									0003723: RNA binding
②	Xl.20056.1.S1_a_at	−6.17049	−1.300266	−1.1079731	−1.3633791	mettl7a	Methyltransferase like 7A	NM_001092436	0008152: metabolic process
		down	down	down	down				0008168: methyltransferase activity
③	Xl.19782.1.A1_s_at	−6.9813504	−1.8626844	−1.3669766	1.3013937	dpm1	Dolichyl-phosphate mannosyltransferase polypeptide 1, catalytic subunit	NM_001095996	0006506: GPI anchor biosynthetic process
		down	down	down	up				0035268: protein amino acid mannosylation
									0035269: protein amino acid O-linked mannosylation
									0005789: endoplasmic reticulum membrane
									0004169: dolichyl-phosphate-mannose-protein mannosyltransferase activity
									0004582: dolichyl-phosphate beta-D-mannosyltransferase activity
④	Xl.6592.1.A1_at	−1.3107206	4.32105	2.4610617	8.463081	lamb1	Laminin subunit beta-1-like (LOC100495516)	XM_002933094	
		down	up	up	up				
⑤	Xl.318.1.S1_at	1.1725949	−1.132029	1.4655828	4.5488386	grem1	Gremlin 1	NM_001090277	0005576: extracellular region
		up	down	up	up				0005615: extracellular space
									0005125: cytokine activity
⑥	Xl.12934.1.A1_at	1.4139103	1.6579978	−1.3287517	1.1197512	bcor	BCL6 co-repressor-like 1	NM_001142070	
		up	up	down	up				
⑦	Xl.15257.1.A1_at	3.5753617	−2.833272	−1.2125118	−1.9222058	MGC130975	Hypothetical protein MGC130975	NM_001096245	
		up	down	down	down				
⑧	Xl.15847.2.A1_at	−1.095607	−4.3573484	−4.801005	−2.5903695	rhno1	RAD9-HUS1-RAD1 interacting nuclear orphan 1	BC170052	
		down	down	down	down				
⑨	Xl.4605.1.A1_at	1.0614563	−1.1746231	10.257936	−1.5519288		EST	BM261602	
		up	down	up	down				

### cDNA cloning from *R. pirica* tadpoles and quantitative real-time PCR

cDNA fragments from *R. pirica* tadpoles were selected on the basis of the results of the cross-species microarray. Mixed total RNA (1 μg) purified from the tail parts of tadpoles exposed to different predation threats were used for cDNA synthesis using 10 pmol of anchored T17ADP primer (5′-GAG TCG ACT CGA GAA TTC T_17_-3′) and Superscript™III (Invitrogen). The selected cDNA fragments were produced by PCR using gene specific primers and KOD FX Neo (TOYOBO). The amplification procedure was performed according to the manufacturer’s protocol. The gene specific primers were designed using information obtained from homology searches of cDNA databases of *X. tropicalis*, *X. laevis*, and two or three other species (NCBI). Each cDNA fragment was cloned into pCRII-TOPO (Invitrogen), and sequencing was carried out using BigDye terminator version 3.1 and an ABI 3100 DNA sequencer (Applied Biosystems). Quantitative real time PCR was performed using Rotor Gene Q MDx 5plex HRM (QIAGEN). In the qPCR, 18S ribosomal RNA obtained from *R. pirica* was used as the internal control. qPCR was performed using Rotor-Gene SYBR® Green PCR Kit (QIAGEN) as described in the manufacturer’s protocol. For each sample, duplicate qPCR amplifications were performed. A 40-cycle program with a hot start was used; the PCR cycles consisted of 5 seconds at 95°C for denaturation, and 10 seconds at 60°C for annealing and elongation. The qPCR primers for *ELAV-like1*(LC011860) and 18S (LC011910) are shown in Additional file 3: Table S1.

## Results

### Discriminant analysis in a cross-species microarray

**Figure 2:**
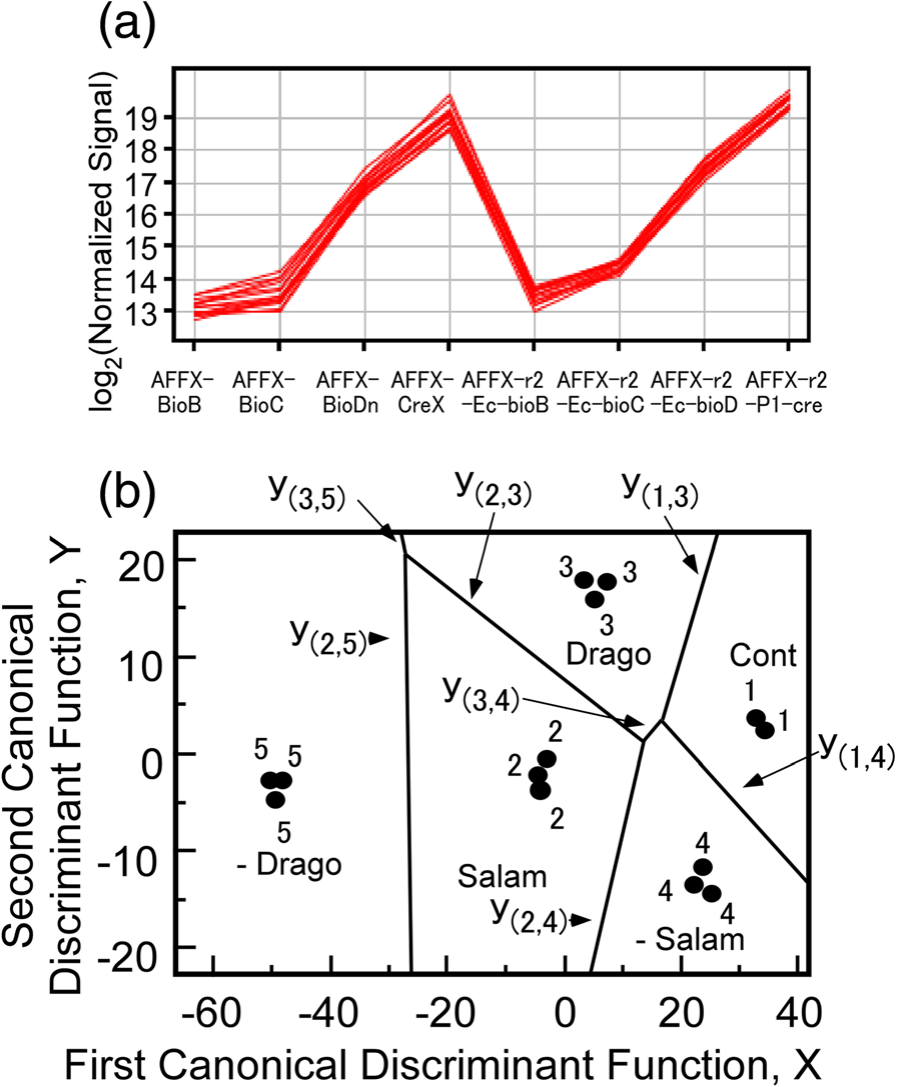
**Discriminant analysis using** ***Xenopus*** **genome array. (a)** Success of hybridization was checked using control genes spotted on the *Xenopus* genome array. AFFX-BioB to AFFX-r2-P1-cre are internal positive controls of *Xenopus* genome array. The analysis was performed using array chips that were hybridized using the same conditions. **(b)** The territorial map based on canonical discriminant functions.

Microarray hybridization was performed as described in Figure 1. The symbols Cont to −Salam in Figure 1 indicate the comparative design of the microarray analysis, which was performed in triplicate. Hybridization conditions were checked using control genes; the chip from control 1 was eliminated from the analysis owing to hybridization failure (Figure 2a).

Ten genes, X|.5177.1.S1_at, X|.318.1.S1_at, X|.12934.1.A1_at, X|.4605.1.A1_at, X|.21688.1.A1_at, X|.20056.1.S1_a_at, X|.6592.1.A1_at, X|.15257.1.A1_at, X|.19782.1.A1_s_at and X|.15847.2.A1_at., were selected by ANOVA of the microarray data from the 5 treatment groups (the control and four different predator conditions) in the present experiment (Figure 1); multiple comparison tests were then performed. For the validation of the selection, discriminant analysis of the cross-species microarray was carried out. In order to avoid the singularity of the variance covariance matrix of the within groups, 9 genes were selected. The first 2 discriminant functions give 97% of the total discrimination using all the discriminant functions, which is sufficient to discriminate among the 5 groups.

The canonical discriminant functions (1) and (2) are:

1 $$ \begin{array}{l}x=-6.973-8.135X\left|.\mathrm{5177.1}.S1\_ at+7.910X\right|.\mathrm{318.1}.S1\_ at-32.407X\Big|.\mathrm{12934.1}.A1\_ at\\ {}\kern3em -6.363X\left|.\mathrm{4605.1}.A1\_ at+19.777X\right|.\mathrm{20056.1}.S1\_a\_ at-0.103X\Big|.\mathrm{6592.1}.A1\_ at\\ {}\kern3em -2.832X\left|.\mathrm{19782.1}.A1\_s\_ at+10.719X\right|.\mathrm{15847.2}.A1\_ at+19.323X\Big|.15257.A1\_ at\end{array} $$

2 $$ \begin{array}{l}y=-0.498+0.708X\left|.\mathrm{5177.1}.S1\_ at+9.184X\right|.\mathrm{318.1}.S1\_ at+0.671X\Big|.\mathrm{12934.1}.A1\_ at\\ {}\kern3em -2.060X\left|.\mathrm{4605.1}.A1\_ at+7.312X\right|.\mathrm{20056.1}.S1\_a\_ at-0.923X\Big|.\mathrm{6592.1}.A1\_ at\\ {}\kern3em -0.569X\left|.\mathrm{19782.1}.A1\_s\_ at+0.512X\right|.\mathrm{15847.2}.A1\_ at+0.188X\Big|.15257.A1\_ at\end{array} $$

The centroid of each group is (35.116, 2.626), (−3.883, −2.388), (5.311, 17.126), (24.414, −13.219), (−49.253, −3.269) for the control, larval salamanders, dragonfly larvae, and removal of salamander and removal of dragonfly larvae, respectively. Figure 2b shows the territorial map based on the two canonical discriminant functions.

The scattered points in Figure 2b are the projection points from the nine genes by the two canonical discriminant functions and the numbers 1 to 5 indicate the five treatment groups. The seven lines, i.e., *y*_(*i*,*j*)_, in Figure 2b indicate the boundary lines between the groups *i* and *j*. For instance, the boundary line between groups 2 and 5, i.e., continuous exposure to larval salamanders and removal of the dragonfly larvae, is given by *y*_(2,5)_ = − 51.488*x* − 1370.757.

**Figure 3:**
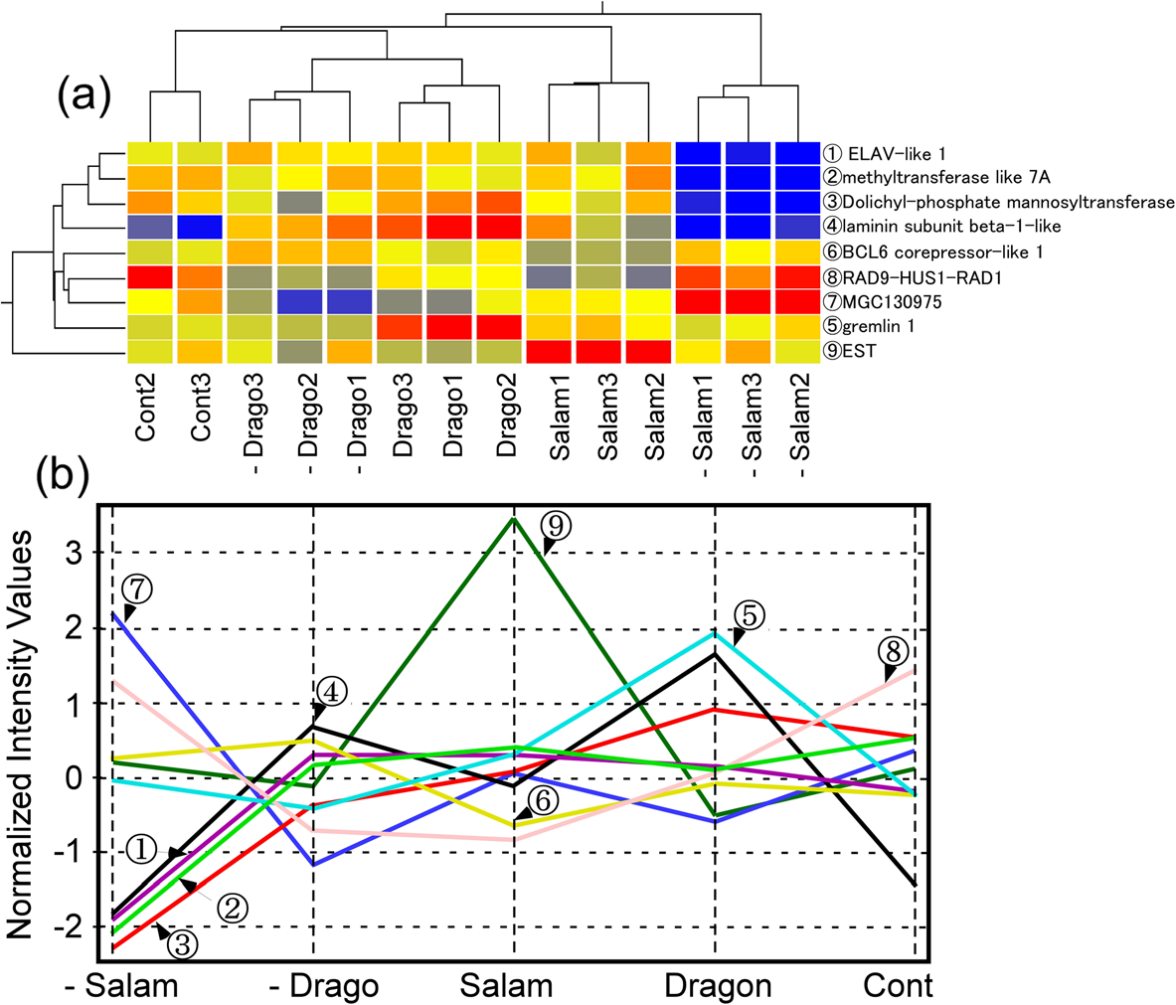
**The nine genes selected by discriminant analysis. (a)** The hierarchical clustering of the nine genes was created using single linkage with Euclidean distance. Numbers 1–3 indicate array chip number. Abbreviations as in Figure 1. **(b)** Averaged gene expression profile obtained by the discriminant analysis.

The nine genes selected by this discriminant analysis are summarized in Table 1 and also shown in the hierarchical combined tree in Figure 3a. Clusters were created using single linkage method with Euclidean distance, and the clusters indicated clear separation of experimental condition such as control, −Drago and so on. This validates the selection method for the nine genes. Further, the same separation was also obtained using 2 other clustering methods: group average method with Euclidean distances and Ward’s method with squared Euclidean distances. The results indicated that expression profiles of the 9 genes are robust.

The identities of three of the nine genes (nos. 7, 8, and 9) are unknown, the others were identified as *ELAV-like 1*, *methyltransferase like 7A*, *dolichyl-phosphate mannosyltransferase*, *laminin subunit beta-1-like*, and *gremlin 1* (Figure 3b).

### Pathway analysis of the nine genes selected by discriminant analysis

**Figure 4:**
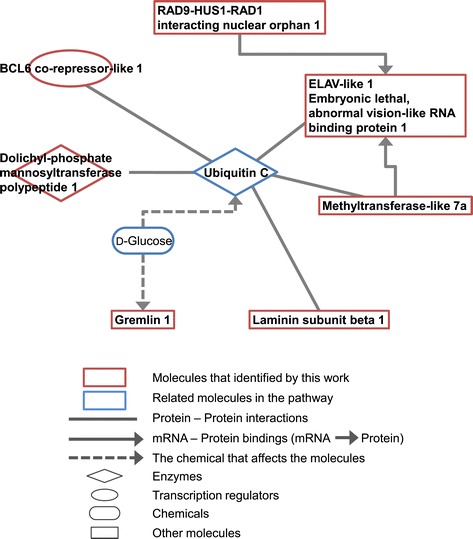
**Pathway analysis of nine genes selected by discriminant analysis.**

The nine genes selected by discriminant analysis were subjected to an IPA pathway analysis using the corresponding human gene. The pathway analysis showed that five of the genes had a protein-protein interaction through ubiquitin C. There was also an mRNA-protein interaction among ELAV-like1, methyltransferase 7A and a hypothetical protein (RAD9-HUS1-RAD1) (Figure 4). The interaction of these proteins belongs to one of the pathways involved in developmental disorders, hereditary disorders and neurological diseases in humans. The postulated interactions regarding these diseases are summarized in Additional file 4: Table S2.

### Screening of specific genes induced by exposure to a predator

**Figure 5:**
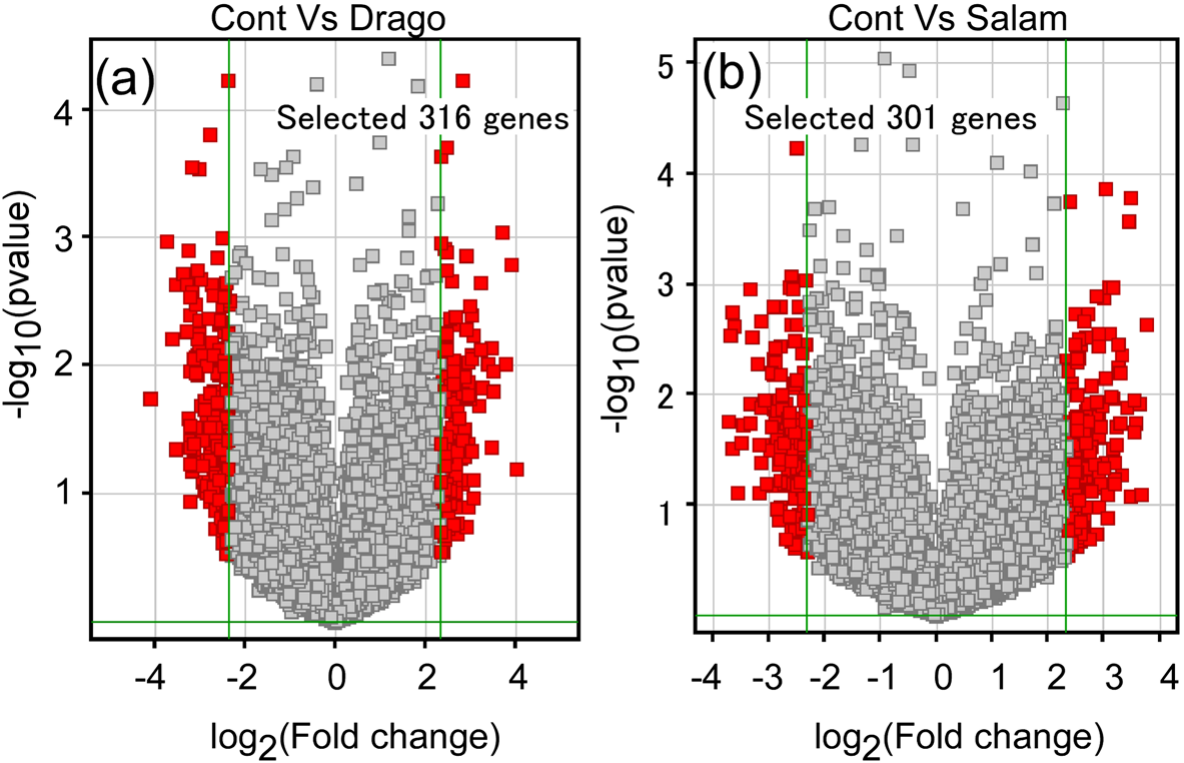
**Screening of predator-induced genes showing a greater than 5 fold difference compared to control.** The selected genes are depicted by volcano plotting, and the threshold change for gene screening was set as ‘more than 5 fold change compared to control’. In total, 316 and 301 genes respectively were identified as induced by dragonfly larvae **(a)** and larval salamanders **(b)**. Fold change is expressed as log_2_X in the X axis.

**Figure 6:**
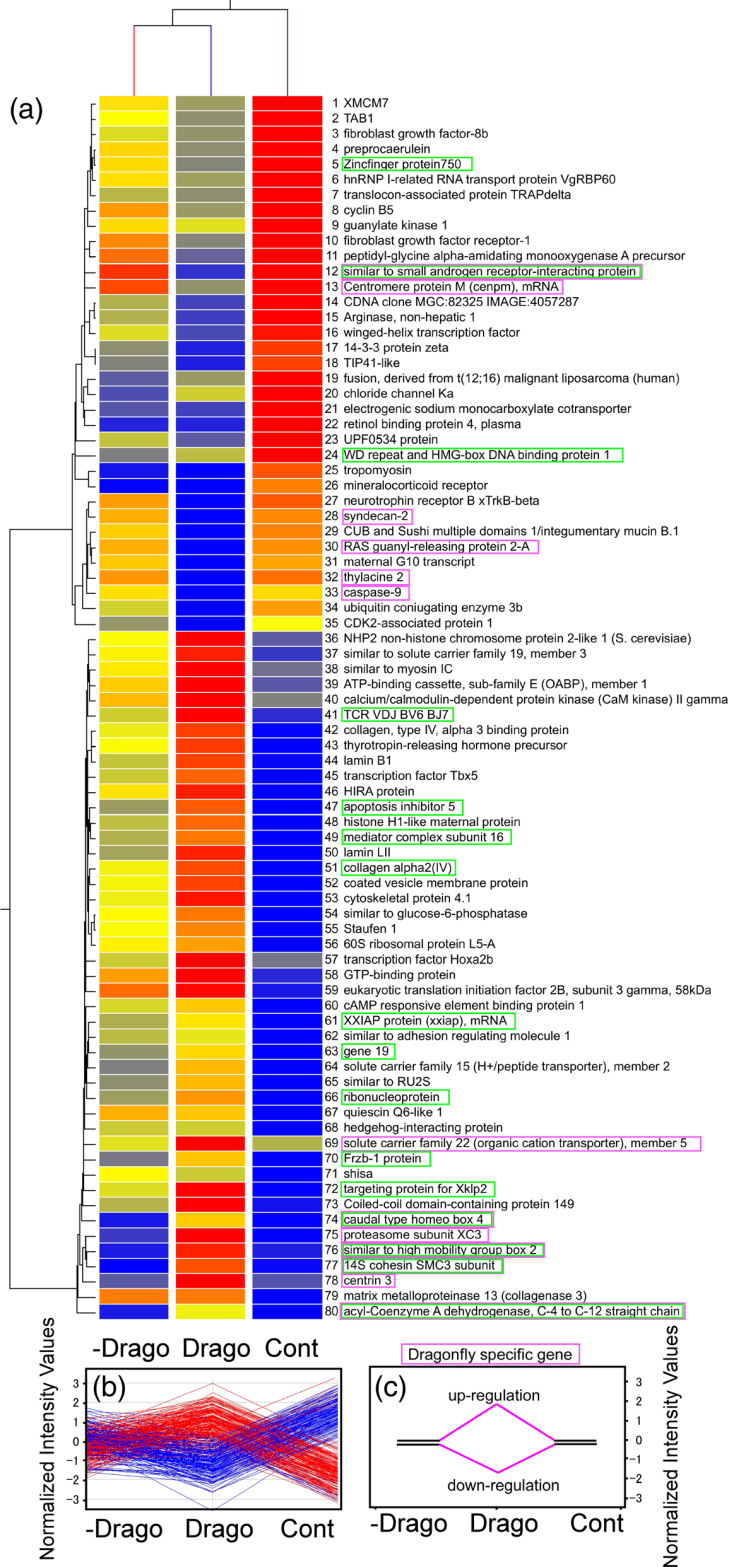
**Hierarchical clustering of 80 known genes showing greater than 5 fold difference compared to control that were induced by dragonfly larvae, and selection of dragonfly specific genes. (a)** Expression profiles of the genes using hierarchical clustering by single linkage with Euclidean distance. **(b)** Expression profiles of the 80 genes and **(c)** procedure used for selection of predation-threat responsive genes. Genes enclosed by pink boxes are dragonfly-specific, and those enclosed in green boxes are those commonly observed after the salamander treatment.

**Figure 7:**
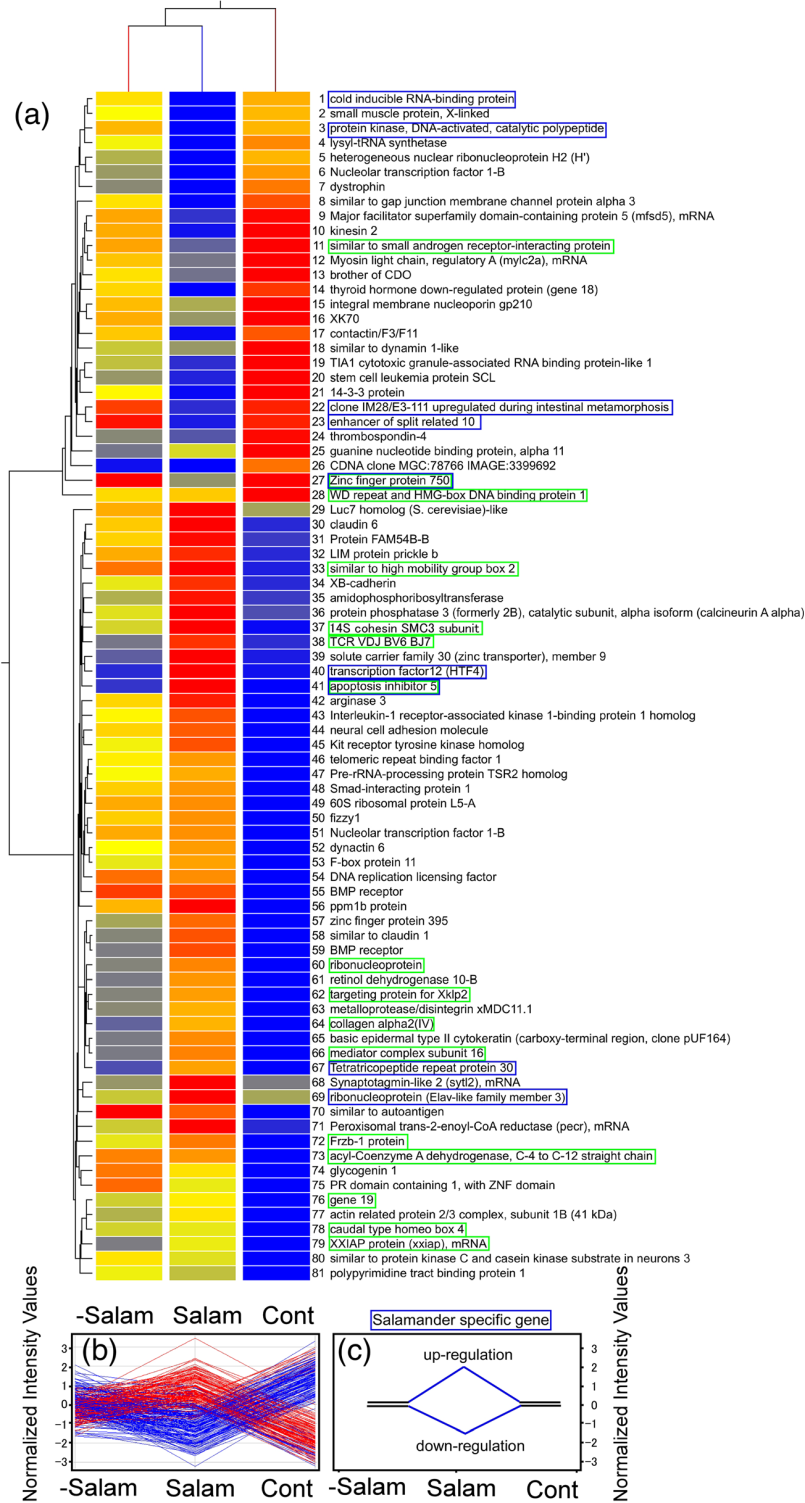
**Hierarchical clustering of 81 known genes showing greater than 5 fold difference to control that were induced by larval salamanders, and the selection of salamander-specific genes. (a)** Expression profiles of the genes using hierarchical clustering by single linkage with Euclidean distance. **(b)** Expression profiles of the 81 genes and **(c)** procedure for selection of predation-threat responsive genes. Genes surrounded by blue boxes are salamander-specific and genes surrounded by green boxes are commonly observed after the dragonfly treatment.

Standard methods were used to determine fold changes in expression of genes following exposure to a predator. We set a threshold change of over 5 fold compared to the control and identified 316 and 301 genes that were induced by exposure to dragonfly larvae or larval salamanders, respectively (Figure 5a and b). These data are also described in Additional file 5: Table S3 and Additional file 6: Table S4. Of these genes, the identities of only 80 and 81 genes, respectively, were known and these were selected as predator-responsive genes. The expression profiles of these genes were compared by a hierarchical clustering analysis using single linkage with Euclidean distance. This analysis indicated that gene #72 (*targeting protein for Xklp2*) showed an 11.5 fold increase in expression and gene #23 (*UPF0534 protein*) showed an 11.3 fold decrease in expression compared to control after exposure to a dragonfly larva (Figure 6). Gene #72 (*Frzb-1 protein*) showed a 12.7 fold increase in expression and gene #21 (*14-3-3 protein*) a 10.9 fold decrease compared to control after exposure to a larval salamander (Figure 7).

**Figure 8:**
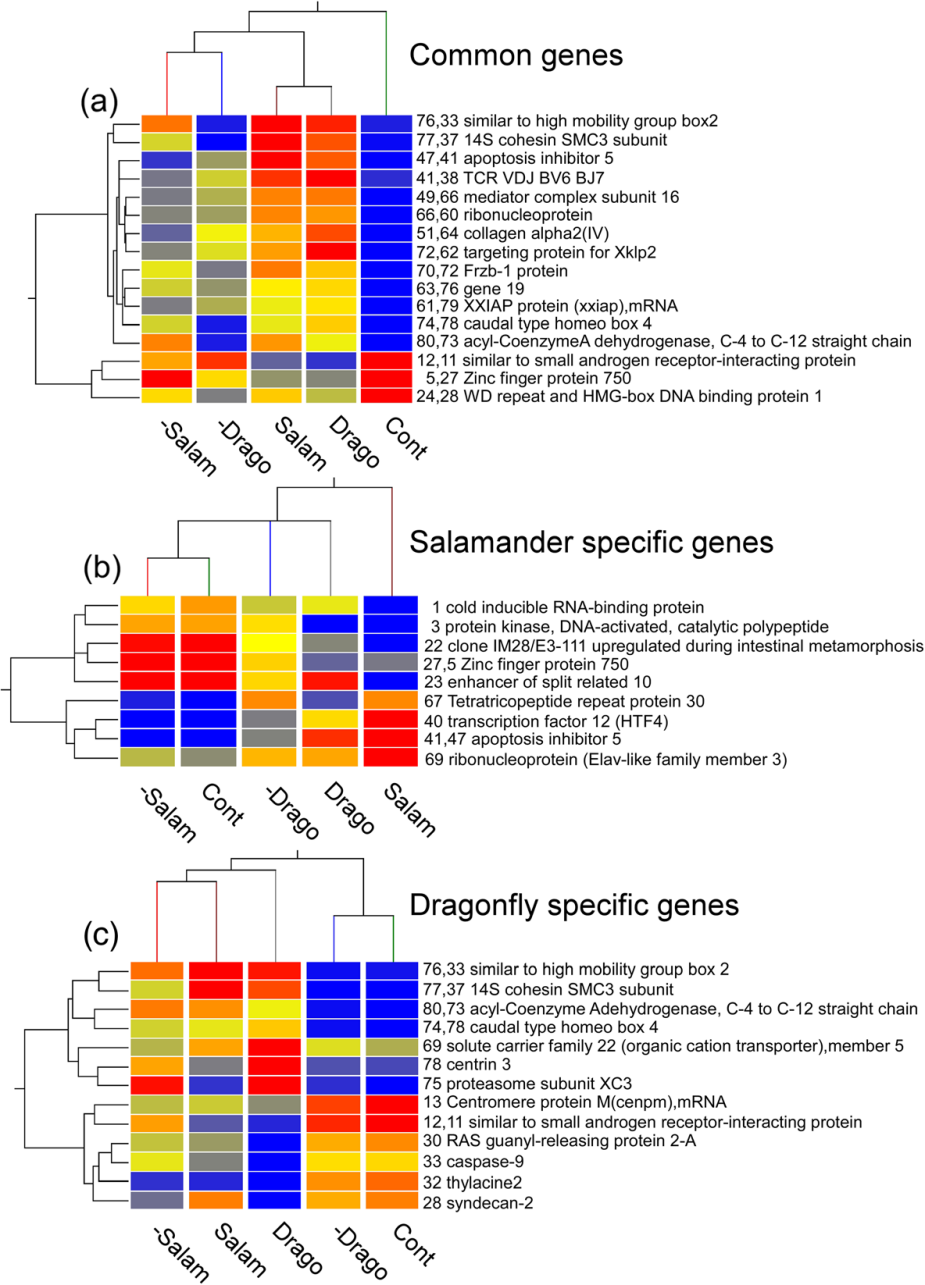
**Hierarchical clustering analysis of common and specific genes induced by predators.** Expression profiles of common and specific genes are depicted by hierarchical clustering using single linkage with Euclidean distance for the five treatment groups. **(a)** Commonly expressed genes selected as showing greater than 5 fold difference to control. The numbers in the gene title (from left to right) in common genes indicate dragonfly and salamander, respectively. **(b)** Salamander specific genes. The numbers in the gene title (from left to right) indicate salamander and dragonfly, respectively. **(c)** Dragonfly specific genes. The numbers in the gene title (from left to right) indicate dragonfly and salamander, respectively.

We also identified predator-responsive genes in the treatment groups in which the predators were removed after 4 days (Figures 6 and 7). The expression profiles of genes showing more than 5 fold changes in expression are shown in Figures 6b and 7b. Selection of genes that are predator-specific can be achieved using the procedure illustrated in Figures 6c and 7c. Thus, for example, the expression level of a predation-threat responsive gene might return to the control level upon removal of the predation threat. Selection of genes that show this pattern of expression should identify those that are predator-specific. Using this approach, we obtained 13 and 9 genes that were specifically responsive to a predation threat by dragonfly and larval salamanders, respectively. These genes are indicated by the pink and blue boxes in Figures 6a and 7a, respectively. A further 16 genes were common to both dragonfly larvae and larval salamanders (Figures 6 and 7); these genes are indicated by the green boxes. The results from hierarchical cluster analyses using single linkage with Euclidean distance of the common and predation-threat responsive genes are summarized in Figure 8a.

In these analyses, the continuous predator exposure groups and the removal of predator groups each formed clusters, and the control formed an outer cluster.

Salamander and dragonfly-specific genes are also summarized (Figure 8b and c). Among those that were real salamander-specific were gene #1 (*cold inducible RNA-binding protein*), gene #3 (protein kinase), gene #22 (*cloneIM28/E3-111*), gene #23 (*enhancer of split related 10*), gene #40 (*transcription factor 12*), gene #67 (*tetratricopeptide repeat protein 30*), and gene #69 (ribonucleoprotein: Elav-like family member 3) (Figure 8b). Although these 9 genes showed similar patterns, *cold inducible RNA-binding protein*, *enhancer of split related 10*, and *tetratricopeptide repeat protein 30* showed opposite expression patterns between Salam and Drago treatment groups. With regard to the dragonfly-specific genes in Figure 8c, gene #78 (*centrin 3*), gene #75 (*proteasome subunit XC3*), and gene #28 (*syndecan-2*) showed the opposite expression patterns in the Salam and Drago treatment groups. These genes might have a role in the different phenotypic responses to the different predators.

### cDNA cloning and quantitative real-time PCR

Eight genes from *R. pirica* tadpoles showed E-values for their sequence data of less than 10^−72^ compared with to *X. laevis*. These data suggesting that these *R. pirica* cDNA sequences were similar to those of *X. laevis*. Furthermore, qPCR analysis of ELAV-like1 likewise showed a similar tendency to that observed in the cross-species microarray using a uniformly most powerful invariant test. The E-values of the sequence data are given in Additional file 7: Table S5 and the statistical analyses of the qPCR are presented in Additional file 8: Figure S2 and Additional file 9.

## Discussion

### Nine genes identified in the discriminant analysis

Predator-induced phenotypic plasticity results in morphological adaptations that confer protection against predators. The alterations to the tail in tadpoles in the presence of either the top predator (dragonfly larvae) or the intermediate predator (larval salamanders) may provide protection through enabling an increased swimming speed to escape from the former or by prevention against swallowing by the latter. It is a reasonable presumption that the phenotypic changes in the predation-related effects are mediated by stress responsive genes.

To determine the identities of the stress responsive genes and to characterize their function, we made use of a *Xenopus laevis* gene chip to screen for functional genes. The use of this cross-species microarray approach was due to the unavailability of a large scale microarray from *Rana pirica*. Although cross-species microarrays suffer from signal reduction, they have nevertheless been used successfully in comparative evolutionary and ecological studies, and for gene-expression profiling of many species that lack representative microarray platforms [31].

In total, nine genes were identified here as predation-responsive, and their functions have been determined through database searches. The ELAV (embryonic lethal, abnormal vision)-like 1 (HuR) protein has various roles in different cellular mechanisms including apoptosis [34,35], oncogenesis [36], cellular responses to stress [37], and the stabilization of several cellular mRNAs [38]. ELAV affects cell fate by regulating the stability and translation of mRNAs that encode cell stress response proteins [37] and stress-induced cell death proteins [35] and also determine responses to DNA damaging agents such as UVC and actinomycin D [39]. Although the available information indicates that HuR is highly related to stress conditions, a dramatic change in *ELAV-like 1* (*HuR*) gene expression (about a 3.3 fold decrease compared with control) was observed following the removal of the larval salamanders (Figure 2 and Table 1). Since the gene showed an increased expression under the other experimental conditions, including removal of the dragonfly larvae (Table 1), then it is likely that the gene is not involved in a response to predation stress here.

*Methyltransferase-like 7a* was down-regulated in all treatments compared with controls (Figure 2 and Table 1). In particular, a 6.2 fold reduction was observed following removal of the larval salamanders. The gene is a *methyltransferase* and may function in histone methylation or other metabolic processes. To date, however, there are no detailed descriptions of the precise function of this gene. Interestingly, expression of the gene is altered in post-traumatic stress disorder and shows a significant decrease in human patients [40]. As described earlier, dragonfly larvae are top predators and very efficient killers of tadpoles when they are kept in the same aquarium. Although larval salamanders also predate tadpoles, they are gape-limited and restricted in the size of the prey they can swallow [10,17]. Thus, the difference between the predation threat posed by the dragonfly and salamander is simply one of relative effectiveness. In the present study, some of the tadpoles were bitten by larval salamanders and a few were killed; however, as the dragonfly larvae were caged, then no tadpoles were harmed in these treatment groups. Therefore, in the salamander treatment groups, it is possible that the tadpoles were showing a stress response to the threat of predation.

Laminins are large, heterotrimeric glycoproteins that are major components of both the intestinal extracellular matrix and the basal lamina [41]. These glycoproteins are involved in cell adhesion [42], neurite outgrowth [43], the formation of epithelial structures [44], and are especially important for the formation of an initial polymeric scaffold of cell-attached matrix via interactions with integrins, lutheran, agrin, dystroglycan, and other components. Laminins, type IV collagen and nidogens are thought to constitute the central basement membrane scaffolding [45]. Thus, laminins clearly interact with other proteins to contribute to cell adhesion and, therefore, presumably promote basement membrane anchorage. Interestingly, we found here that *laminin subunit beta-1-like* was increased 8.4 fold in tadpoles continuously exposed to dragonfly larvae and 4.3 fold in those in which the larvae were removed (Table 1). Moreover, *laminin B1* and *laminin LII* were also increased 5.6 and 7.5 fold compared to the control in the treatment group with continuous exposure to dragonfly larvae (Figure 6). However, in the treatment group with continuous exposure to larval salamanders, a 2.46 fold increase compared to control was observed; in the group in which the larval salamanders were removed a 1.3 fold decrease was found. It will be of interest to determine whether the altered patterns of laminin expression are related to changes in cell-cell adhesion in tadpoles with heightened tails.

Gremlin 1 is a bone morphogenetic protein (BMP) antagonist, and has an important role in regulating BMPs during lung, limb, and kidney development as well as during neural crest cell differentiation [46,47]. Gremlin 1 is overexpressed in various human tumors [48,49], and induces cell migration, proliferation, and invasion through induction of a fibroblast-like morphology and decreased E-cadherin expression [50]. Gremlin also down-regulates chondrogenesis and programmed cell death [51]. In our experiments, dragonfly treatment induced greatest expression of the gremlin gene (Figure 3), down-regulation of caspase-9, and increased expression of apoptosis inhibitor 5 (Figure 6). In the salamander treatment, a slight increase in gremlin gene expression was observed, and apoptosis inhibitor 5 was also clearly increased (Figure 7).

Under hypoxic conditions, HIF-lα isoforms are stabilized and induce HIF-dependent transcription of *Vegf, Greml*, and *Noggin* [52]. Gremlin 1 expression is increased in the walls of the small vessels of the pulmonary circulation in vivo during the development of hypoxic pulmonary hypertension [53]. In our microarray experiment, two types of *HIF1a* gene were used as a probe, and the largest increases in expression (3 fold and 1.3 fold compared to control) were observed in the dragonfly treatment groups; similarly, the highest increase in expression of *gremlin 1* (4.5 fold compared to control) occurred in a dragonfly treatment (Table 1). Exposure to a predation threat from larval salamanders also induced an approximately 1.5 fold increase in the expression of *gremlin 1*. These results may indicate that predation stress involves hypoxic reactions and programmed cell death in vivo.

*BCL6 corepressor-like 1* (*BCoR-L1*) is a transcription corepressor and functional studies have shown that it can bind to class II histone deacetylases, and interact with the CTBP1 (C terminal binding protein 1) corepressor to repress expression of E-cadherin [54]. *BCoR-L1* displays homology to several proteins involved in DNA damage repair pathways and in transcription regulation (*BCoR*) [54]. It is also involved in the regulation of proliferation and apoptosis [55]. Interestingly, we found higher expression of the gene following the removal of the dragonfly larvae or larval salamanders (1.7 and 1.4 fold higher expression than control, respectively; Table 1); only continuous exposure to larval salamanders caused a decrease in gene expression.

The remaining three genes were of unknown identity, although gene #9 showed the highest increase in expression in the salamander treatment group among the nine genes. Therefore, the function of gene #9 might be important in the formation of the bulgy morph in response to the threat of salamander predation.

The IPA analysis showed that 7 of the selected 9 genes were connected directly or indirectly to ubiquitin C (Figure 4). Therefore, these genes involve the ubiquitin-proteasome system. As described above, ELAV-like1 is a multifunctional protein, has been reported to be in the ubiquitin-proteasome pathway in the regulation and function of HuR (an ELAV-like1 protein) [56], and is also related to ubiquitination in predator-induced morphological changes. Further, since ELAV-like 1 protein binds methyltransferase-like 7A and RAD9-HUS1_RAD1 mRNA, then expression of *ELAV-like1* may be regulated by these two genes.

A combination of discriminant and IPA analyses identified the involvement of the ubiquitin-proteasome system, suggesting that ubiquitination may have a role to play in predator-induced morphological changes.

Genes showing 5 fold differences in expression compared to control.

Overall, 80 and 81 genes were selected as responsive to the dragonfly larvae and larval salamander predation treatments, respectively (Figures 6 and 7). In these experiments, we were seeking to identify predation-threat responsive genes and 9 were selected as salamander-specific (Figure 8b). Two of these genes, zinc finger protein 750 and *apoptosis inhibitor 5*, were also included in the set of common genes. Three genes, *cold inducible RNA-binding protein* (5.7 fold reduction compared to control), *enhancer of split related 10* (5.9 fold reduction), and *tetratricopeptide repeat protein 30* (6.6 fold increase) showed the reverse expression profile in tadpoles exposed to larval salamanders compared to dragonfly larvae. Therefore, these three genes seemed to be further specific for the salamander treatment.

Cold inducible RNA-binding protein (*CIRP*) is induced by cellular stress such as cold shock [57], UV irradiation [58], oxidative stress or hypoxia [59], and inhibition of neural apoptosis [60]. Down-regulation of *CIRP* results in decreased cell proliferation [61]. The *CIRP* homologue in *Xenopus,* called *XCIRP-1* [62], was used as the probe on this Xenopus microarray. Down-regulation of *XCIRP-1* inhibits expression of adhesion molecules such as *αE-* and *β-catenin*, *C-* and *E-cadherin* in Xenopus embryos [63]. As we found that exposure to larval salamanders enhances radical production in tadpoles compared to controls (unpublished data), the down-regulation of *XCIRP-1* in the salamander treatment group was unexpected. Expression of *C-cadherin* was decreased in all treatment groups except control, and was 2.5 and 2.0 fold lower than control in the salamander and dragonfly treatment groups, respectively. *α(E)-catenin* (Xl.6961.1.S1_at) also showed the greatest decrease (2.4 fold compared to control) in the salamander treatment group.

Interestingly, muscle related genes (gene #2, *small muscle protein*; gene #7, *dystrophin*; gene #12, *myosin light chain*) were down-regulated in tadpoles exposed to larval salamanders (Figure 7). In the dragonfly treatment groups, gene #25 (*tropomyosin*) was down-regulated; however, gene #51 (*collagen alpha 2*), gene #62 (*adhesion regulating molecule*), gene #38 (*similar to myosin IC*) showed increased expression. These results point to a complex response, possibly related to the structural changes in tail morphology, in tadpoles subjected to a predation threat.

Gene #23 (*enhancer of split related 10*) was down-regulated only by the salamander treatment (Figure 8b). The *Xenopus* homologue of this gene is an isoform of the human gene *Hairy* and *enhancer of split homologue 5* (*Hes5*) [64], and this negatively regulates cell differentiation during embryogenesis [65]. It is not presently clear whether the predator-induced morphological changes to tadpole tail morphology involve a cell differentiation process.

Tetratricopeptide repeat protein 30 has a role in tubulin glycosylation and glutamylation for maintaining cilia structure and motility [66]. Cilia and basal bodies play important roles in many physiological processes, including cell and fluid movements, sensory perception and development [67], and ion channels that function as chemo-, osmo-, or mechanosensors [68,69]. This gene showed opposite responses in tadpoles of the dragonfly and salamander treatment groups (Figure 8b): a 6.6 fold increase was seen in the salamander treatment group, while a 6.5 fold increase occurred following the removal of the dragonfly larvae compared to control. In the salamander treatment, tadpoles maintain their osmotic pressure by uptake of Na and Cl ions to increase the amount of bodily fluids for bulgy morph formation [30]. Possibly, non-motile or sensory cilia may be involved in this process.

With regard to dragonfly-specific genes, *centrin 3*, proteasome subunit, and *syndecan-2* showed different expression patterns between salamander and dragonfly treatment groups. Interestingly, *centrin 3* also showed a somewhat opposite reaction in the salamander and dragonfly treatment groups (Figure 8c). Centrin is conserved in a variety of eukaryotes [70], and is involved with inhibition of cell proliferation [71]. However, it is unclear whether the up-regulation of *centrin3* in tadpoles exposed to dragonfly larvae is associated with a change in the rate of cell proliferation.

*Syndecans* (types 1–4) are a family of heparan sulfate-bearing transmembrane proteoglycans involved in cell adhesion, mobility and growth factor interactions [72-74]. The *syndecan-2* ectodomain promotes focal adhesion and stress fiber formation in fibroblasts in a distinctly different pattern to fibronectin and independently of heparin sulfate requirement [75]. *Syndecan-2* also controls assembly of laminin and fibronectin into a fibrillar matrix [76]. However, *syndecan-2* down-regulation impairs angiogenesis in human microvascular endothelial cells, and reduces spreading and adhesion of endothelial cells, thereby enhancing their migration but also impairing the formation of capillary-like structures [77]. The data in the microarray analysis showed an approximately 6.2 and 3.2 reduction in *syndecan-2* expression compared with the control in the dragonfly and removal of salamander treatment groups, respectively. With regard to bulgy morph formation, it is reasonable to expect the tadpoles to develop a vascular system for maintaining body fluids. However, down-regulation of angiogenesis might be required when the salamander predation threat is removed. Exposure to dragonfly larvae predation may also induce down-regulation of angiogenesis to cause edema; the dragonfly is a top predator and, therefore, tadpoles may need to increase their swimming ability to avoid capture. In addition, as described above, the highest expression of *HIF* genes was observed in the dragonfly treatment group. This result might also be connected to the impairment of angiogenesis.

## Conclusions

In this study, we have identified some key genes involved in the adaptation of tadpole bodies in response to specific predators. These predation-threat responsive genes seem to function in producing morphological changes that depend on the nature of the predation threat; the selected genes might also include causal or associated genes for adaptation to predators. Recently, numerous reports have shown that signal transduction in gene expression pathways are connected directly or indirectly. Therefore, predator-specific genes might be responsible not only for responses to predation threat but also to many types of stress. Therefore, the common genes listed in Figure 8a might have the potential to be related or indirect genes induced by both predators. However, the single gene depicted as salamander and dragonfly specific genes (Figure 8b and c) might have the potential to be a causal gene for the specific responses to each predator.

Although the threat of predation by larval salamanders or by dragonfly larvae induces the formation of a heightened tail, gene expression profiles in tadpoles exposed to these predators were different suggesting functional differences in the modified tail tissue. It will be of interest to determine what factors stimulate these different patterns of expression of key genes.

## Additional files

Additional file 1:
**Supplementary information 1.** Statistical investigation of Rana pirica tadpole cDNAs with microarrays from *X.laevis.*
Additional file 2: Figure S1. Relationships of the e-values obtained after using *Rana pirica* tadpole cDNAs to probe microarrays from *Xenopus laevis* and *Xenopus tropicalis.* Nine e-values were zero and were omitted from the figure. Additional file 3: Table S1. Primers for qPCR of ELAV-like1 and 18S obtained from *R. pirica*. Additional file 4: Table S2. Summarized references on IPA analysis for selected genes by discriminant analysis. Additional file 5: Table S3. Dragonfly larvae induced 316 genes that showed greater than 5 fold difference to control. Additional file 6: Table S4. Larval salamanders induced 301 genes that showed greater than 5 fold difference to control. Additional file 7: Table S5. E-values for sequence data from *R. pirica* tadpoles. Additional file 8: Figure S2. Quantitative PCR of ELAV-like 1 gene expression normalized by Rana pirica 18S ribosomal RNA. Additional file 9:
**Supplementary information 2.** Comparison between the population mean vector from the microarray and that from real-time PCR.

## References

[1] Spitze K (1992). Predator-mediated plasticity of prey life history and morphology: Chaoborus americanus predation on Daphnia pulex. Am Nat.

[2] Trussell GC (1996). Phenotypic plasticity in an intertidal snail: the role of a common crab predator. Evolution.

[3] Schoeppner NM, Relyea RA (2005). Damage, digestion, and defence: the roles of alarm cues and kairomones for inducing prey defences. Ecol Lett.

[4] Pigliucci M (2001). Phenotypic plasticity.

[5] West-Eberhard MJ (2003). Developmental plasticity and evolution.

[6] Bronmark C, Miner JG (1992). Predator-induced phenotypical change in body morphology in Crucian Carp. Science.

[7] Tollrian R (1995). Predator-induced morphological defenses - costs, life-history shifts, and maternal effects in Daphnia-Pulex. Ecology.

[8] Tollrian R, Harvell CD (1999). The ecology and evolution of inducible defenses.

[9] Weisser WW, Braendle C, Minoretti N (1999). Predator-induced morphological shift in the pea aphid. Proc R Soc Lond Ser B Biol Sci.

[10] Kishida O, Nishimura K (2004). Bulgy tadpoles: inducible defense morph. Oecologia.

[11] Jarrett JN (2009). Predator-induced defense in the Barnacle Chthamalus fissus. J Crustac Biol.

[12] Gilbert JJ (2009). Predator-specific inducible defenses in the rotifer Keratella tropica. Freshw Biol.

[13] McCollum SA, Leimberger JD (1997). Predator-induced morphological changes in an amphibian: predation by dragonflies affects tadpole shape and color. Oecologia.

[14] Van Buskirk J, McCollum SA, Werner EE (1997). Natural selection for environmentally induced phenotypes in tadpoles. Evolution.

[15] Kishida O, Trussell GC, Mougi A, Nishimura K (2010). Evolutionary ecology of inducible morphological plasticity in predator–prey interaction: toward the practical links with population ecology. Popul Ecol.

[16] Van Buskirk J, Relyea RA (1998). Selection for phenotypic plasticity in Rana sylvatica tadpoles. Biol J Linn Soc.

[17] Kishida O, Nishimura K (2005). Multiple inducible defences against multiple predators in the anuran tadpole. Rana pirica Evol Ecol Res.

[18] Michimae H, Wakahara M (2002). A tadpole-induced polyphenism in the salamander Hynobius retardatus. Evolution.

[19] Michimae H, Nishimura K, Wakahara M (2005). Mechanical vibrations from tadpoles’ flapping tails transform salamander’s carnivorous morphology. Biol Lett.

[20] Kishida O, Trussell GC, Nishimura K (2007). Geographic variation in a predator-induced defense and its genetic basis. Ecology.

[21] Kishida O, Trussell GC, Nishimura K, Ohgushi T (2009). Inducible defenses in prey intensify predator cannibalism. Ecology.

[22] Van Buskirk J (2002). A comparative test of the adaptive plasticity hypothesis: relationships between habitat and phenotype in anuran larvae. Am Nat.

[23] Kishida O, Trussell GC, Nishimura K (2009). Top-down effects on antagonistic inducible defense and offense. Ecology.

[24] Pigliucci M (2003). Phenotypic integration: studying the ecology and evolution of complex phenotypes. Ecol Lett.

[25] Stearns SC, Magwene P (2003). The naturalist in a world of genomics: an American society of naturalists symposium paper. Am Nat.

[26] Thomas MA, Klaper R (2004). Genomics for the ecological toolbox. Trends Ecol Evol.

[27] Relyea RA (2005). The heritability of inducible defenses in tadpoles. J Evol Biol.

[28] Mori T, Hiraka I, Kurata Y, Kawachi H, Kishida O (2005). Genetic basis of phenotypic plasticity for predator-induced morphological defenses in anuran tadpole, Rana pirica, using cDNA subtraction and microarray analysis. Biochem. Biophys. Res. Commun.

[29] Mori T, Kawachi H, Imai C, Sugiyama M, Kurata Y, Kishida O (2009). Identification of a novel uromodulin-like gene related to predator-induced bulgy morph in anuran tadpoles by functional microarray analysis. PLoS One.

[30] Mori T, Kitani Y, Ogihara J, Sugiyama M, Yamamoto G, Kishida O (2012). Histological and MS spectrometric analyses of the modified tissue of bulgy form tadpoles induced by salamander predation. Biol Open.

[31] Bar-Or C, Czosnek H, Koltai H (2007). Cross-species microarray hybridizations: a developing tool for studying species diversity. Trends Genet.

[32] Hsu JC (1996). Multiple comparisons: theory and methods.

[33] Everitt B (1974). Cluster analysis, vol. section 3.1 and chapter 4.

[34] Lal A, Kawai T, Yang X, Mazan-Mamczarz K, Gorospe M (2005). Antiapoptotic function of RNA-binding protein HuR effected through prothymosin alpha. Embo J.

[35] Mazroui R, Di Marco S, Clair E, von Roretz C, Tenenbaum SA, Keene JD (2008). Caspase-mediated cleavage of HuR in the cytoplasm contributes to pp 32/PHAP-I regulation of apoptosis. J Cell Biol.

[36] Lopez de Silanes I, Lal A, Gorospe M (2005). HuR: post-transcriptional paths to malignancy. RNA Biol.

[37] Gorospe M (2003). HuR in the mammalian genotoxic response: post-transcriptional multitasking. Cell Cycle.

[38] Brennan CM, Steitz JA (2001). HuR and mRNA stability. Cell Mol Life Sci.

[39] Wang W, Furneaux H, Cheng H, Caldwell MC, Hutter D, Liu Y (2000). HuR regulates p21 mRNA stabilization by UV light. Mol Cell Biol.

[40] Yehuda R, Cai G, Golier JA, Sarapas C, Galea S, Ising M (2009). Gene expression patterns associated with posttraumatic stress disorder following exposure to the World Trade Center attacks. Biol Psychiatry.

[41] Timpl R, Brown JC (1994). The laminins. Matrix Biol.

[42] Nakatsuji N (1986). Presumptive mesoderm cells from Xenopus laevis gastrulae attach to and migrate on substrata coated with fibronectin or laminin. J Cell Sci.

[43] Lander AD, Fujii DK, Reichardt LF (1985). Purification of a factor that promotes neurite outgrowth: isolation of laminin and associated molecules. J Cell Biol.

[44] Sorokin L, Sonnenberg A, Aumailley M, Timpl R, Ekblom P (1990). Recognition of the laminin E8 cell-binding site by an integrin possessing the alpha 6 subunit is essential for epithelial polarization in developing kidney tubules. J Cell Biol.

[45] Yurchenco PD, Amenta PS, Patton BL (2004). Basement membrane assembly, stability and activities observed through a developmental lens. Matrix Biol.

[46] Lu MM, Yang H, Zhang L, Shu W, Blair DG, Morrisey EE (2001). The bone morphogenic protein antagonist gremlin regulates proximal-distal patterning of the lung. Dev Dyn.

[47] Shi W, Zhao J, Anderson KD, Warburton D (2001). Gremlin negatively modulates BMP-4 induction of embryonic mouse lung branching morphogenesis. Am J Physiol Lung Cell Mol Physiol.

[48] Namkoong H, Shin SM, Kim HK, Ha SA, Cho GW, Hur SY (2006). The bone morphogenetic protein antagonist gremlin 1 is overexpressed in human cancers and interacts with YWHAH protein. BMC Cancer.

[49] Sha G, Zhang Y, Zhang C, Wan Y, Zhao Z, Li C (2009). Elevated levels of gremlin-1 in eutopic endometrium and peripheral serum in patients with endometriosis. Fertil Steril.

[50] Kim M, Yoon S, Lee S, Ha SA, Kim HK, Kim JW (2012). Gremlin-1 induces BMP-independent tumor cell proliferation, migration, and invasion. PLoS One.

[51] Merino R, Rodriguez-Leon J, Fau-Macias D, Macias Fau D, Ganan Y, Ganan-Fau Y (1999). The BMP antagonist Gremlin regulates outgrowth, chondrogenesis and programmed cell death in the developing limb. Development.

[52] Genetos DC, Toupadakis CA, Raheja LF, Wong A, Papanicolaou SE, Fyhrie DP (2010). Hypoxia decreases sclerostin expression and increases Wnt signaling in osteoblasts. J Cell Biochem.

[53] Cahill E, Costello CM, Rowan SC, Harkin S, Howell K, Leonard MO (2012). Gremlin plays a key role in the pathogenesis of pulmonary hypertension. Circulation.

[54] Pagan JK, Arnold J, Hanchard KJ, Kumar R, Bruno T, Jones MJ (2007). A novel corepressor, BCoR-L1, represses transcription through an interaction with CtBP. J Biol Chem.

[55] Jepsen K, Rosenfeld MG (2002). Biological roles and mechanistic actions of co-repressor complexes. J Cell Sci.

[56] Abdelmohsen K, Fau SS, Yang X, Fau YX, Lal A, Fau LA (2009). Ubiquitin-mediated proteolysis of HuR by heat shock. EMBO J.

[57] Fujita J (1999). Cold shock response in mammalian cells. J Mol Microbiol Biotechnol.

[58] Yang C, Carrier F (2001). The UV-inducible RNA-binding protein A18 (A18 hnRNP) plays a protective role in the genotoxic stress response. J Biol Chem.

[59] Wellmann S, Buhrer C, Moderegger E, Zelmer A, Kirschner R, Koehne P (2004). Oxygen-regulated expression of the RNA-binding proteins RBM3 and CIRP by a HIF-1-independent mechanism. J Cell Sci.

[60] Li S, Zhang Z, Xue J, Liu A, Zhang H (2012). Cold-inducible RNA binding protein inhibits H(2)O(2)-induced apoptosis in rat cortical neurons. Brain Res.

[61] Artero-Castro A, Callejas FB, Castellvi J, Kondoh H, Carnero A, Fernandez-Marcos PJ (2009). Cold-inducible RNA-binding protein bypasses replicative senescence in primary cells through extracellular signal-regulated kinase 1 and 2 activation. Mol Cell Biol.

[62] Peng Y, Kok KH, Xu RH, Kwok KH, Tay D, Fung PC (2000). Maternal cold inducible RNA binding protein is required for embryonic kidney formation in Xenopus laevis. FEBS Lett.

[63] Peng Y, Yang PH, Tanner JA, Huang JD, Li M, Lee HF (2006). Cold-inducible RNA binding protein is required for the expression of adhesion molecules and embryonic cell movement in Xenopus laevis. Biochem Biophys Res Commun.

[64] Lamar E, Kintner C (2005). The Notch targets Esr1 and Esr10 are differentially regulated in Xenopus neural precursors. Development.

[65] Liu J, Lu WG, Ye F, Cheng XD, Hong D, Hu Y (2010). Hes1/Hes5 gene inhibits differentiation via down-regulating Hash1 and promotes proliferation in cervical carcinoma cells. Int J Gynecol Cancer.

[66] Pathak N, Austin CA, Drummond IA (2011). Tubulin tyrosine ligase-like genes ttll3 and ttll6 maintain zebrafish cilia structure and motility. J Biol Chem.

[67] Scholey JM (2003). Intraflagellar transport. Annu Rev Cell Dev Biol.

[68] Perkins LA, Hedgecock EM, Thomson JN, Culotti JG (1986). Mutant sensory cilia in the nematode Caenorhabditis elegans. Dev Biol.

[69] Scholey JM, Anderson KV (2006). Intraflagellar transport and cilium-based signaling. Cell.

[70] Salisbury JL (1995). Centrin, centrosomes, and mitotic spindle poles. Curr Opin Cell Biol.

[71] Middendorp S, Kuntziger T, Abraham Y, Holmes S, Bordes N, Paintrand M (2000). A role for centrin 3 in centrosome reproduction. J Cell Biol.

[72] Oh ES, Couchman JR (2004). Syndecans-2 and −4; close cousins, but not identical twins. Mol Cells.

[73] Tkachenko E, Rhodes JM, Simons M (2005). Syndecans: new kids on the signaling block. Circ Res.

[74] Alexopoulou AN, Multhaupt HA, Couchman JR (2007). Syndecans in wound healing, inflammation and vascular biology. Int J Biochem Cell Biol.

[75] Whiteford JR, Behrends V, Kirby H, Kusche-Gullberg M, Muramatsu T, Couchman JR (2007). Syndecans promote integrin-mediated adhesion of mesenchymal cells in two distinct pathways. Exp Cell Res.

[76] Klass CM, Couchman JR, Woods A (2000). Control of extracellular matrix assembly by syndecan-2 proteoglycan. J Cell Sci.

[77] Noguer O, Villena J, Lorita J, Vilaro S, Reina M (2009). Syndecan-2 downregulation impairs angiogenesis in human microvascular endothelial cells. Exp Cell Res.

