# Supplementary material for: Gene expression profiles in Rana pirica tadpoles following exposure to a predation threat
Source: BMC Genomics. 2015 Apr 2;16(1):258. doi: 10.1186/s12864-015-1389-4 (PMC4403775; doi:10.1186/s12864-015-1389-4)
Supplement: Additional file 1: — Supplementary information 1. Statistical investigation of Rana pirica tadpole cDNAs with microarrays from X.laevis. [file 12864_2015_1389_MOESM1_ESM.doc]

**Additional file 1. Supplementary information 1**

The approach adopted in our study was to search a *Xenopus* microarray for homologues of RNAs expressed in tail tissue of *Rana pirica* tadpoles. The two commercially available microarrays were developed from *Xenopus laevis* and *X. tropicalis.* We generated a set of e-values between the sequences of *X. laevis* genes and *Rana pirica* cDNAs; a second set was similarly generated for *X. tropicalis.* The e-values are an estimate of the similarity between two sequences and lie between 0 and 1. An e-value of 0 indicates a perfect match, and increases in e-value indicate increasing dissimilarity. A blast analysis (NCBI) was applied using 1020 cDNAs from *Rana pirica* with a threshold set at 1e-10. In total, 294 and 345 hits were obtained from *X. laevis* and *X. tropicalis;* 275 queries hit both *Xenopus sp,* and also retrieved same gene name from both *Xenopus* databases*.* Therefore, these 275 e-values were compared using a paired t-test between *X. laevis* and *X. tropicalis.* Validation of the t-test is described below.

Let $Eval(Rana,a,i;laevis,b,j)$ be the e-value between two nucleotides sequences *of R. pirica* and *X. laevis*, i.e. the sequence of gene $a$ (e.g. query X) of $i$-th individual of *Rana pirica* and the sequence of gene $b$ (gene name such as enolase 1) of $j$-th individual of *X. laevis*. Assume that $Eval(Rana,a,i;laevis,b,j)$ is a random variable which is independently identically distributed as normal with mean $Eval(Rana,a;laevis,b)$ for gene $a$ of *R. pirica* and for gene $b$ of *X. laevis,* and an unknown constant variance $\sigma^{2}$. Suppose that $Eval(Rana,laevis)$ is the mean of $Eval(Rana,a;laevis,b)$ for the group of those 275 genes. $Eval(Rana,a;tropicalis,b)$ and $Eval(Rana;tropicalis)$ are defined similarly too. The mean of $Eval(Rana,a,i;laevis,b,j)-Eval(Rana,a,i;tropicalis,b,h)$ for $i$, $j$ and $h$, and also for the group of those 275 genes is independently identically distributed as normal with the mean $Eval(Rana;laevis)-Eval(Rana;tropicalis)$ where genes $b$’s are the same for both species *X. laevis* and *X. tropicalis*. We then have a t-test for testing the mean $Eval(Rana;laevis)-Eval(Rana;tropicalis)=0$ against $Eval(Rana;lavis)-Eval(Rana;tropicalis)\neq0$ for at least one of the pairs of $a$ and $b$ for 275 genes. The P-value of this t-test was 0.0613, so the null hypothesis was not rejected at 5% significance level (Supplementary Fig.1). Therefore both *X. laevis* and *X. tropicalis* gave the same population mean e-values for *Rana pirica*. This result indicates there is no statistical difference in the microarray platforms of *X. laevis* and *X. tropicalis* for detection of mRNAs from *Rana pirica*.
