# Supplementary figures and images for: Gene expression profiles in Rana pirica tadpoles following exposure to a predation threat
Source: BMC Genomics. 2015 Apr 2;16(1):258. doi: 10.1186/s12864-015-1389-4 (PMC4403775; doi:10.1186/s12864-015-1389-4)

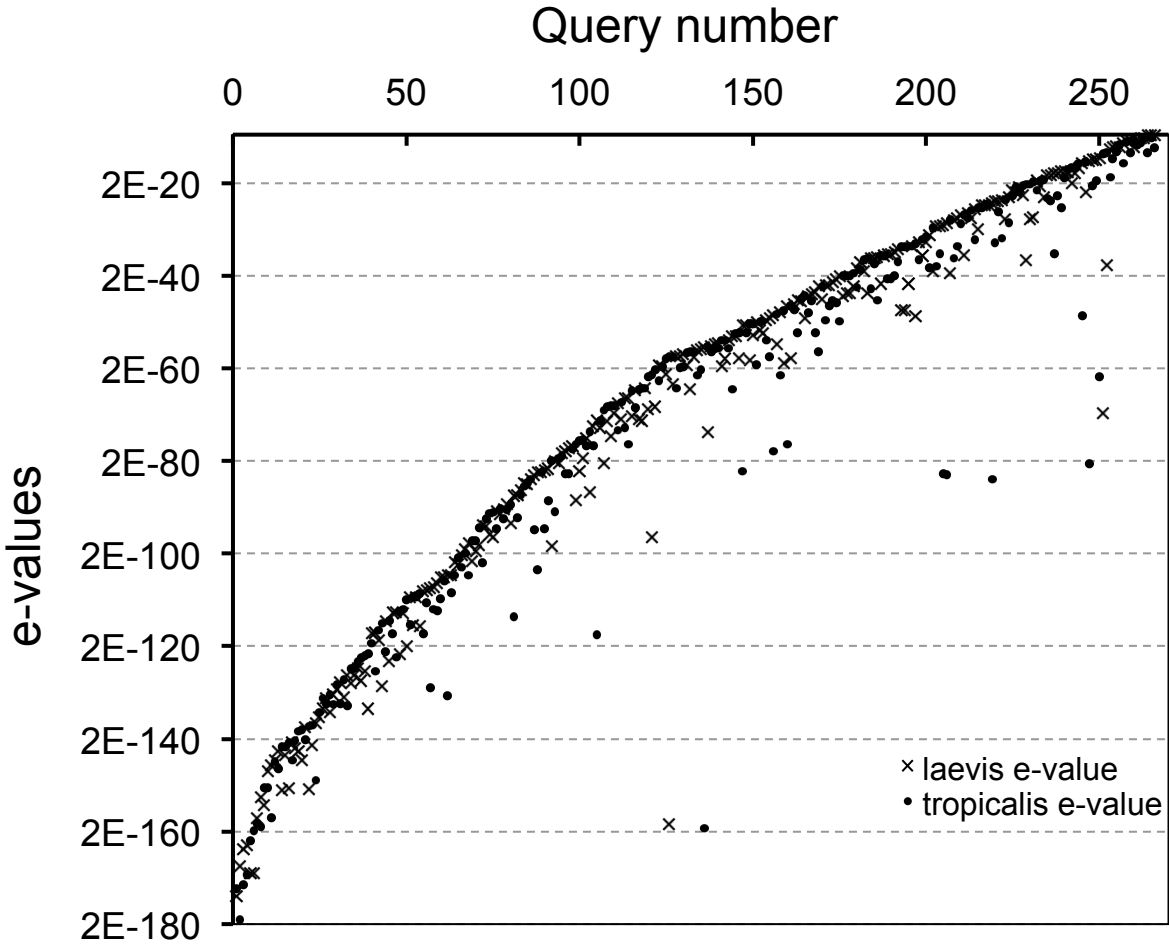

Supplement: Additional file 2: Figure S1. — Relationships of the e-values obtained after using Rana pirica tadpole cDNAs to probe microarrays from Xenopus laevis and Xenopus tropicalis. Nine e-values were zero and were omitted from the figure. [file 12864_2015_1389_MOESM2_ESM.pdf]

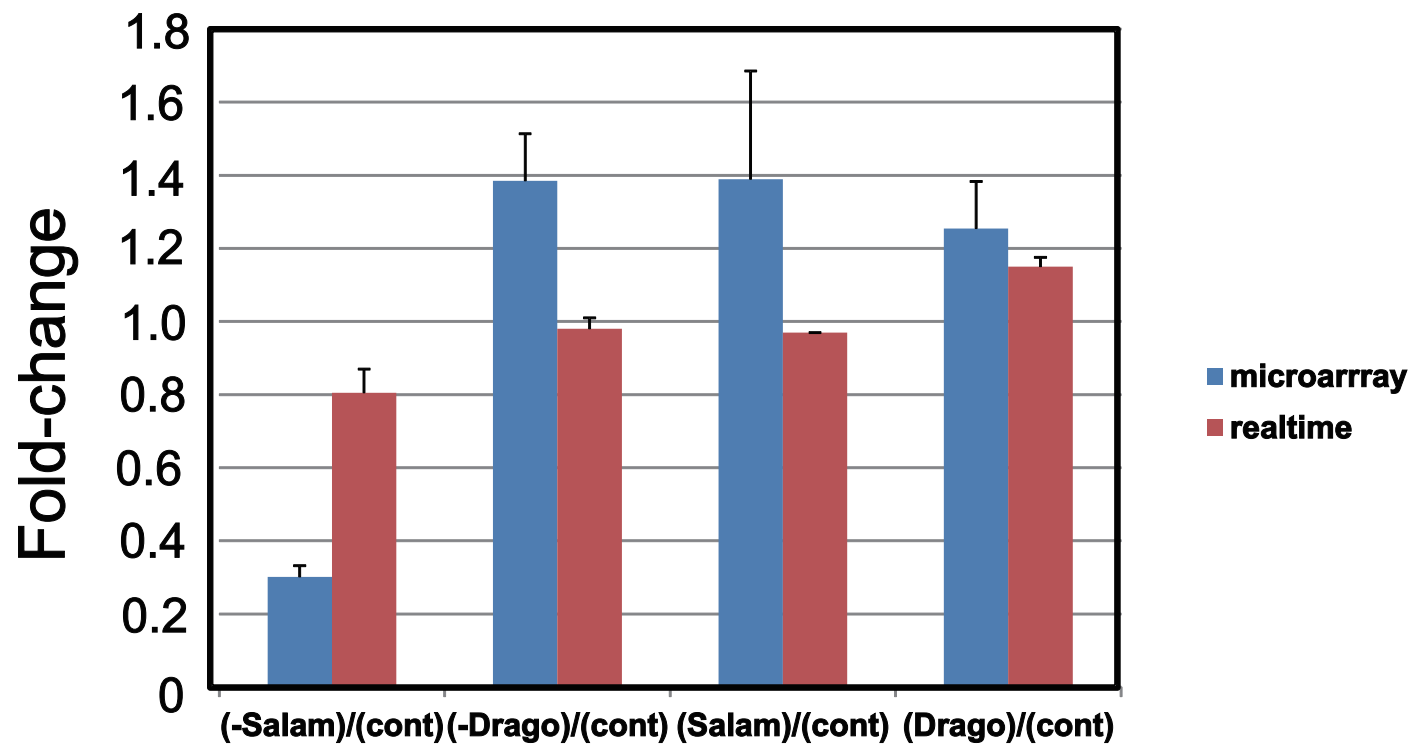

Supplement: Additional file 8: Figure S2. — Quantitative PCR of ELAV-like 1 gene expression normalized by Rana pirica 18S ribosomal RNA. [file 12864_2015_1389_MOESM8_ESM.pdf]
