# Supplementary material for: Gene expression profiles in Rana pirica tadpoles following exposure to a predation threat
Source: BMC Genomics. 2015 Apr 2;16(1):258. doi: 10.1186/s12864-015-1389-4 (PMC4403775; doi:10.1186/s12864-015-1389-4)
Supplement: Additional file 9: — Supplementary information 2. Comparison between the population mean vector from the microarray and that from real-time PCR. [file 12864_2015_1389_MOESM9_ESM.doc]

**Additional file 9. Supplementary information 2.**

**Supplementary Information 2**

Consider the comparison between the population mean vector from the microarray and that from real-time PCR. The sample mean vector from the microarray and that from real-time PCR were, respectively, (1.46, 0.30, 1.27, 1.40) and (0.97, 0.81, 1.16, 0.98), where the 4 elements of the mean vectors were Salamander/Control, -Salam/Control, Dragonfly/Control and　　　　　 -Drago/Control, respectively. As we do not have enough observations to calculate the inverse matrix of the variance-covariance matrix, we can only statistically test 3 elements at most for the equality of the two mean vectors, instead of the 4 elements, i.e., the choice is 3 of 4. It is clear that repeating tests is not a good idea as it increases the rejection region (see multiple comparisons). The analysis showed that the nearest two sample mean elements were for Dragonfly/Control, i.e. 1.27 and 1.16, so we removed Dragonfly/Control for testing the equality of the population mean vectors. This particular element was removed because the test is the equivalence of the two population mean vectors against inequality of the mean vectors for Salamander/Control, -Salam/Control, and -Drago/Control. If we cannot reject the null hypothesis, excluding the element of Dragonfly/Control which gave the closest sample means, it would be the best assurance for the result of testing two population mean vectors under these circumstances, because we would be testing the worst scenario within the 4 combinations.

For testing two population mean vectors, we use Hotelling’s $T^{2}$.

where$\bar{X}$, $\bar{Y}$, $S$, $n_{x}$ and $n_{y}$ are the sample mean vectors of real-time PCR, that of the microarray, pooled variance-covariance matrix, the sample size of real-time PCR and that of the microarray, respectively.

$\frac{n_{x}+n_{y}-k-1}{(n_{x}+n_{y}-2)k}T^{2}$ is approximately distributed as $F_{k,n_{x}+n_{y}-k-1}$ under the null hypothesis where $k$ is the dimension of the vector, i.e. 3 in this case. This is a uniformly most powerful invariant test, i.e., there is no better test than this.

We have

$T^{2}=170.632$, $\frac{n_{x}+n_{y}-k-1}{(n_{x}+n_{y}-2)k}T^{2}=18.959$ and p-value=0.16688. We therefore cannot reject the null hypothesis, i.e. the equivalence of the two population mean vectors, at a significance level of 5%.
